# Redefining Medical Professionalism in Taiwan: A Qualitative Study of Societal Expectations and Educational Implications

**DOI:** 10.5334/pme.1828

**Published:** 2025-11-03

**Authors:** Ching-Yi Lee, Sze-Yuen Yau, Mi-Mi Chen, Hung-Yi Lai

**Affiliations:** 1 Department of Neurosurgery, Chang Gung Memorial Hospital, Linkou, Taiwan; 2 Chang Gung University College of Medicine, Taoyuan, Taiwan; 3 (CG-MERC) Chang Gung Medical Education Research Centre, Linkou, Taiwan

## Abstract

**Background:**

Global frameworks of medical professionalism are often grounded in Western liberal values, which may misalign with ethical expectations in non-Western contexts. In Taiwan, where Confucian traditions shape clinical interactions, professionalism is rooted in relational ethics, family-centered care, and emotional labor. This study explored how professionalism is culturally defined and negotiated among key stakeholder groups in Taiwanese healthcare.

**Methods:**

A qualitative, constructivist-interpretivist design guided the study. Eleven focus groups were conducted with 78 participants from a major teaching hospital and surrounding community in northern Taiwan, including practicing physicians (n = 25), medical students (n = 20), and members of the public (n = 33). A constructivist-interpretivist paradigm informed data collection and thematic analysis using Braun and Clarke’s six-phase approach. Coding was conducted in Mandarin and triangulated by an interdisciplinary research team with clinical, sociological, and educational expertise.

**Results:**

Eleven subthemes were identified and organized into three themes: (1) humanistic and relational values (e.g., empathy, moral character, emotional labor); (2) professional expectations (e.g., communication, accountability, lifelong learning); and (3) cultural tensions (e.g., service orientation, family obligations versus patient autonomy). Stakeholders expressed divergent views of professionalism, particularly between public expectations and formal curricular ideals.

**Discussion:**

Findings underscore the need for culturally responsive professionalism education in Confucian-influenced contexts. Integrating relational ethics, emotional attunement, and family-centered values into curricula, assessment, and faculty development may better align training with societal expectations. This study offers a contextualized framework for professionalism that reconciles global standards with local moral landscapes.

## Background

Medical professionalism is widely regarded as a cornerstone of health professions education and practice, frequently articulated through global frameworks such as the Accreditation Council for Graduate Medical Education (ACGME) and the CanMEDS Physician Competency Framework [1,2]. These models typically emphasize principles such as individual autonomy, altruism, integrity, and self-regulation, positioning professionalism as a moral contract between the profession and society [3,4,5]. However, these frameworks are grounded in Western liberal individualist traditions and may not fully capture the moral logics and social expectations that characterize professionalism in non-Western societies [6,7]. In East Asian contexts, where Confucian relational ethics, familial obligations, and collectivist values remain influential, medical professionalism may be understood and enacted differently, raising questions about the universal applicability of Western professionalism models [7,8,9].

In Taiwan, Confucian moral philosophy continues to shape social relationships, institutional structures, and ethical expectations within healthcare [10,11]. Core Confucian values such as filial piety (xiao, 孝), benevolence (ren, 仁), and ritual propriety (li, 禮) emphasize relational obligations, hierarchical harmony, and emotional restraint [10,12]. These values influence how physicians interact with patients and families, often prioritizing family-centered decision-making, moral character, and affective labor over autonomous decision-making or rights-based discourse [8,13]. For example, studies have documented culturally normative practices such as nondisclosure of terminal diagnoses to preserve familial harmony, which may conflict with Western conceptions of truth-telling and informed consent [14,15]. Taiwan is home to a diverse population comprising Hoklo, Hakka, Indigenous peoples, and growing numbers of immigrants, each with distinct historical, linguistic, and cultural traditions. These communities may differ in their expectations of medical professionalism, shaped by variations in social norms, communication styles, and healthcare experiences. Recognizing this diversity is critical to situating professionalism within Taiwan’s pluralistic sociocultural context. At the same time, we acknowledge that individual differences—arising from personal values, education, and life experiences—can be as influential as cultural background in shaping one’s understanding of professionalism. These divergences suggest the need for a culturally grounded reappraisal of what constitutes professionalism in East Asian healthcare systems. These groups may differ in linguistic traditions, cultural practices, and health-related values, which can shape their expectations of medical professionalism in distinct ways. As such, any culturally grounded framework for professionalism in Taiwan must be attentive to both shared moral traditions and the pluralism of cultural perspectives within the population.

Although Confucian ethics continue to shape Taiwanese society, medical education has largely imported Western professionalism frameworks without adapting them to local cultural contexts [16,17,18]. Professionalism is often taught as a fixed set of abstract principles or traits, with limited engagement with students’ lived experiences, cultural backgrounds, or the implicit moral messages conveyed through institutional practices [19,20,21]. Scholars have argued that these disconnects between formal curricula and informal or hidden curricula can erode students’ professional ideals and moral sensitivity over time [19,20,22]. Moreover, the social contract underpinning professionalism in Taiwan has been destabilized by broader systemic changes, including the implementation of the National Health Insurance (NHI) system, increased bureaucratic regulation, and shifting public expectations [11,23,24]. These developments have challenged physicians’ autonomy and public trust, further complicating the meaning and enactment of professionalism.

In response, scholars have called for culturally responsive and contextually situated models of professionalism in East Asian medical education [9,25]. Relational autonomy has emerged as a promising theoretical alternative, emphasizing individuals’ embeddedness in social relationships and the moral significance of care, mutual recognition, and communal responsibility [12]. This orientation aligns with the lived moral experience of many Taiwanese physicians and patients, for whom professional identity is constructed not in isolation, but through interdependent roles and emotional labor within families and communities [7,18]. Similarly, recent educational scholarship has underscored the importance of integrating humanistic values such as empathy, compassion, respect, and service into the formal curriculum through reflective practice, narrative medicine, and faculty development [18,21,26].

Yet, few empirical studies in Taiwan have examined how professionalism is defined and negotiated across multiple stakeholder groups. Existing research has largely focused either on educators or trainees, overlooking the perspectives of practicing physicians and the public—voices that are essential for understanding professionalism as a social constructed phenomenon [27,28]. Addressing this gap requires an interpretivist, constructivist approach that attends to the diverse moral imaginaries, value conflicts, and relational dynamics that inform professional identity formation. To this end, we conducted focus groups with three stakeholder groups—practicing physicians, final-year medical students, and members of the public—to explore convergences and divergences in their conceptualizations of professionalism. This triangulated design, grounded in social construction theory, captures professionalism as a negotiated moral and social contract, enabling a more complete account of its culturally situated and co-constructed nature than single-group studies can provide.

## Methods

This study employed a qualitative, interpretivist design grounded in constructivist ontology. We conceptualized professionalism not as a universal attribute, but as a socially constructed and culturally embedded phenomenon, shaped by interpersonal relationships, institutional cultures, and broader societal expectations [27,28]. In alignment with this epistemological stance, we used focus group methodology to elicit multiple, dialogically constructed perspectives from physicians, medical trainees, and members of the public in Taiwan. This approach enabled us to explore how professionalism is co-constructed across social roles, rather than individually defined, or universally prescribed.

### Setting and Participants

The study was conducted at Chang Gung Memorial Hospital, a major tertiary teaching hospital in northern Taiwan, and surrounding community settings. We recruited participants from three stakeholder groups:

1. Practicing physicians: Including attending physicians, residents, and postgraduate year (PGY) trainees, all of whom hold a medical license. (n = 25)
2. Medical Students: Final-year undergraduate medical students undertaking hospital-based clinical clerkship training, who have not yet obtained a medical license. (n = 20)
3. General public: Including patients, family members, and community residents. (n = 33)

Purposive and stratified sampling strategies were employed to ensure representation across gender, age, and professional role. Recruitment was facilitated through flyers and direct invitations distributed in hospital clinics, waiting areas, and local community centers. Inclusion criteria included being over 20 years of age, fluent in Mandarin, and willing to participate in a 90-minute focus group. Physicians and students were drawn from a range of specialties to capture interprofessional variation in perspectives.

### Data Collection

Between July and October 2020, we conducted 11 focus group sessions, each comprising 5 to 8 participants and lasting between 65 and 92 minutes. Sessions were moderated by trained facilitators (one male, one female) with expertise in qualitative interviewing. Where possible, moderator gender was matched to participant group composition to align with local preferences for gender concordance in sensitive discussions. A semi-structured interview guide was developed based on a review of empirical and conceptual literature on professionalism, including the physician–patient relationship and culturally situated professionalism frameworks [17,29]. Open-ended questions were designed to elicit participants’ expectations of physicians, perceptions of professional conduct, and lived experiences related to trust, empathy, and moral behavior in healthcare interactions. The guide was reviewed by two medical sociologists and a physician-educator to ensure cultural appropriateness and linguistic clarity. All focus groups were conducted in Mandarin Chinese. Audio recordings were made with participant consent and transcribed verbatim, yielding 21–36 single-spaced pages per session. Identifying information was redacted during transcription to maintain confidentiality. Prior to participation, individuals completed a demographic questionnaire and provided written informed consent. Ethnic background data were not collected, in accordance with IRB requirements to protect participant confidentiality in small focus group settings. Given the relatively small and close-knit professional communities within the study site, reporting ethnicity in combination with other demographic information could potentially identify individual participants.

### Data Analysis

Thematic analysis followed Braun and Clarke six-phase approach, conducted in an iterative and reflexive manner [30]. Two authors (CYL, MMC) independently read all transcripts multiple times to familiarize themselves with the data and generated initial codes inductively. A third author (HYL) reviewed these codes for completeness and cultural contextual accuracy. All three authors met to discuss and consolidate the coding framework. In the theme development phase, CYL, MMC, and HYL grouped related codes into candidate themes, which were then reviewed and refined in joint meetings with the full author team. YSY cross-checked the revised themes against the original transcripts to ensure that they captured the breadth and depth of participant perspectives. Discrepancies were resolved through discussion until consensus was reached. Throughout the process, the authors maintained analytic memos and engaged in reflexive dialogue to address potential biases and ensure cultural sensitivity. This collaborative approach facilitated a nuanced interpretation of the data that was grounded in the Taiwanese sociocultural context. Data analysis was supported by Atlas.ti (version 9). To enhance the rigor and trustworthiness of the study, we employed multiple strategies: triangulation across disciplinary backgrounds (sociology, clinical medicine, medical education), peer debriefing sessions, and the maintenance of an audit trail documenting analytic decisions. Coding discrepancies were resolved through discussion and consensus. Data saturation was determined when the final two focus groups yielded no substantially new codes, and the thematic patterns had been sufficiently constructed for analytic coherence.

### Ethical Considerations

Ethical approval for the study was granted by the Institutional Review Board of Chang Gung Memorial Hospital (IRB No. 201407781B0). All participants provided written informed consent, including consent for audio recording. They were informed of their right to withdraw from the study at any point without penalty. Data were anonymized during transcription and analysis to ensure confidentiality and ethical integrity throughout the research process.

## Results

The study involved 78 participants across three stakeholder groups (Table 1). Thematic analysis of 11 focus groups yielded three overarching themes that articulate how medical professionalism is constructed, interpreted, and contested in the Taiwanese cultural context: (1) Core Values Underpinning Professionalism, (2) Expected Competencies and Behaviors, and (3) Cultural and Systemic Tensions Shaping Professionalism in Practice. Within each overarching theme, we identified multiple subthemes—eleven in total—that elaborate specific dimensions of participants’ perspectives. Across subthemes, perspectives from the public, physicians, and medical students revealed areas of consensus and divergence, offering a nuanced portrait of professionalism as a dynamic, culturally embedded phenomenon. Together, these findings address our research question by revealing how shared moral principles, culturally shaped expectations, and systemic pressures intersect to define professionalism in Taiwan.

**Table 1:** Participant demographics by stakeholder group.


STAKEHOLDER GROUP	n	GENDER (F/M)

General public	33	22/11

Medical trainees	20	9/11

Physicians (attendings/residents/PGY)	25	2/23

Total	78	33/45


*Ethnic background was not collected, in accordance with IRB guidance, to protect confidentiality in small focus groups and avoid potential identification of participants in sensitive discussions. Notes: Data collection occurred December 2015–May 2016; average session duration ≈ 1.5 hours; number of focus groups—public (6), medical trainees (3), physicians (5).

### Theme 1: Core Values Underpinning Professionalism

#### 1. Humanism-Based Care

Participants frequently described professionalism as rooted in humanistic values—compassion, moral virtue, and emotional sensitivity. Members of the public emphasized that a “good doctor” must first be a good human being; technical skill alone was insufficient. *One participant asserted that “a professional physician must treat patients as if they were their own family members… if you don’t have that heart, you might as well not wear the white coat*” (Public Group 3). For the public, humanism was a moral imperative that defined professional legitimacy.

Physicians and medical students endorsed the importance of humanistic care but underscored the emotional hazards of over-identification with patients. A student explained, “*If we take every case too personally, we would emotionally collapse by the end of the week… we have to find a way to care without burning out*” (Medical Students Group 2). For trainees, sustaining professionalism meant balancing empathy with psychological boundaries to preserve resilience.

This divergence reveals a core tension in the Taiwanese context: the cultural ideal of boundless empathy contrasts with the sustained emotional workload of clinical practice. Professionalism here is described not only as a moral aspiration but also as the skillful management of emotional resources.

#### 2. Family-Centered Empathy

Participants also highlighted the centrality of the family in healthcare interactions. Public participants expressed a strong expectation that physicians involve family members in decision-making, particularly with a serious illness. As one noted, “*the family carries the biggest responsibility for care… they know the patient’s background, and they suffer alongside the patient*” (Public Group 4).

Physicians acknowledged this expectation but recognized its tension with principles of patient autonomy: “What we are taught in ethics class—about patient autonomy and privacy—often doesn’t match what happens in real practice. If we exclude the family, it’s seen as rude… but sometimes the patient’s wishes are different from what the family wants” (Physician Group 3).

These perspectives show how professionalism is interpreted through participants’ views of relational ethics. Public expectations anchor decision-making authority in the family, while physicians described navigating dual obligations—upholding individual autonomy and honoring the family’s sanctioned role.

#### 3. Moral Character and Role Modeling

Professionalism was also closely linked to moral character, with both public and professional groups viewing ethical conduct as a reflection of personal virtue. The public positioned physicians as moral leaders whose influence extends beyond clinical boundaries: “*Physicians have social power, so they must set an example of good behavior… you should never betray that trust, even outside the hospital*” (Public Group 2).

For medical students, professionalism was often learned implicitly through observing senior physicians. One student explained, “*When I see a senior doctor apologize sincerely to a patient for a mistake, it teaches me more about professionalism than any textbook example*” (Medical Students Group 1). Such observations illustrate the strength of the informal curriculum in transmitting professional norms—through everyday behaviors, ethical decision-making, and patient interactions of respected mentors.

This dynamic underscores that cultivating professionalism in Taiwan depends heavily on the quality and consistency of role modeling. The actions of senior physicians become not just examples but active shapers of the next generation’s moral and professional identity.

**Theme 1 Synthesis**. Taken together, the subthemes under Theme 1 illustrate that professionalism in Taiwan is anchored in relational and humanistic values that extend beyond technical skill. Public participants foregrounded compassion, empathy, and moral character as essential qualities of a “good doctor,” while physicians and students described the tensions of enacting these ideals amidst emotional strain and boundary-setting. Family-centered care and role modeling further highlight the moral and relational expectations embedded in the Taiwanese sociocultural context. Collectively, these subthemes suggest that professionalism is judged not only through clinical competence but through relational ethics, affective labor, and moral integrity, forming the cultural baseline for professional legitimacy.

### Theme 2: Expected Competencies and Behaviors

#### 4. Communication Skills

Clear, respectful, and empathetic communication was repeatedly identified as a hallmark of professionalism, though public and professional perspectives revealed a tension between ideals and clinical realities. Public participants emphasized that professionalism was demonstrated not only by what physicians say but how they say it—valuing warmth, patience, and attentiveness as much as factual accuracy. One participant explained, “*A professional physician explains things clearly and speaks with warmth… even if they’re busy, they should make you feel you’ve been heard*” (Public Group 5).

Physicians agreed that good communication was integral to professional care but pointed to the structural constraints of high-volume clinics, where each consultation may be limited to mere minutes. As one remarked, “*We have limited time with each patient… it’s hard to deliver reassurance, context, and emotional support in six minutes*” (Physician Group 1). For many physicians, the challenge lay in balancing the desire for meaningful interaction with the necessity of maintaining efficiency in a pressured system.

These accounts suggest that in Taiwan, communication is framed as both a relational practice and a performance under time pressure, requiring physicians to convey empathy within tight temporal boundaries. The public’s expectation of unhurried dialogue collides with systemic constraints, creating a persistent gap between desired and feasible communication behaviors. This divergence highlights the difficulty of sustaining interpersonal engagement when system constraints and professional ideals are misaligned.

#### 5. Emotional Labor

Participants across all groups viewed the physician’s emotional presence as a critical component of professionalism, though they differed in how they understood and experienced it. Public participants described composure, patience, and emotional availability as non-negotiable qualities: “*A professional physician never shows impatience, even when they are tired or frustrated. The patient should never feel they are a burden*” (Public Group 6). This framing positioned emotional steadiness as a moral obligation—an unwavering sign of respect and care.

Clinical stakeholders, particularly medical students and junior doctors, acknowledged the importance of such composure but revealed its hidden cost. “*After 30 patients in a row, it’s hard to show the same smile, but you have to—otherwise people think you’re cold or rude. It’s draining, and no one teaches you how to recharge*” (Medical Students Group 3). Their accounts framed emotional labor not as effortless virtue but as sustained affective regulation, often performed in the context of physical exhaustion and hierarchical pressure.

The contrast between public expectations and physicians lived experiences underscores that professionalism in Taiwan extends beyond clinical competence into the management of one’s own affective display. While the public reads composure as an authentic expression of care, physicians and students often described it as a disciplined performance that can be emotionally taxing. Many participants noted that strategies for managing this burden are rarely taught, leaving them to find ways of coping on their own.

#### 6. Honesty and Transparency

Truth-telling was identified as a foundational marker of professionalism across all stakeholder groups, but the meaning and enactment of “honesty” diverged sharply. Public participants consistently equated professionalism with full and immediate disclosure: “*A professional physician tells the truth, even if it’s bad news—because trust comes from honesty, not from sugarcoating*” (Public Group 2). In this view, transparency was not simply a communication choice but an ethical absolute, directly tied to trustworthiness and moral integrity.

Physicians, however, described a more nuanced, context-sensitive approach, often shaped by cultural expectations and relational dynamics. As one explained, “*We sometimes soften the message or give it in stages to avoid unnecessary fear or family conflict. It’s not lying—it’s pacing the truth*” (Physician Group 4).

The divergence between these perspectives highlights a potential fault line in professional trust. While the public frames honesty as absolute and immediate, clinicians operationalize it through a lens of relational responsibility, weighing the potential harms of unfiltered truth against the benefits of gradual disclosure. This gap suggests that expectations around truth-telling may be a source of misunderstanding between patients, families, and clinicians.

#### 7. Technical Competence and Lifelong Learning

Across all groups, competence was considered an essential, non-negotiable foundation of professionalism. For members of the public, this expectation was so deeply assumed that it often went unspoken, with attention directed instead toward interpersonal and moral conduct. As one participant noted, “*All physicians should be competent—that’s a given. It’s how they treat people that shows professionalism*” (Public Group 1). This assumption reflects the enduring cultural prestige of the medical profession in Taiwan, where physicians are traditionally viewed as highly trained and inherently capable.

For physicians and medical students, however, competence was not a static credential but a continuous process of development. “Staying updated is part of professionalism. Medicine changes fast; if you stop learning, you stop being professional. It’s a duty to your patients” (Physician Group 5). In this framing, clinical expertise is inseparable from the obligation to engage in lifelong learning—reading new research, mastering novel technologies, and adapting practice in response to evolving evidence.

These contrasting emphases—public assumption versus professional vigilance—reflect different vantage points on the same principle. The public measures competence primarily through visible care quality, assuming underlying technical skill. Professionals, conversely, experience competence as an ongoing, self-renewing commitment that demands sustained intellectual effort. Within Taiwan’s rapidly advancing medical landscape, this distinction reinforces the idea that professionalism is as much about the discipline of continual self-improvement as it is about the mastery of existing knowledge.

#### 8. Accountability and Responsibility

Accountability was described as a core marker of professional integrity, though its meaning diverged between stakeholder groups. Public participants viewed accountability in personal terms, expecting physicians to own their decisions and openly acknowledge mistakes. As one participant explained, “*A professional physician admits when they are wrong. Hiding mistakes is unprofessional—it’s about integrity*” (Public Group 4). In this framing, accountability is a moral stance grounded in honesty and respect for patients’ right to know.

Physicians, however, described accountability within the realities of complex healthcare systems. Errors, they emphasized, are rarely the product of a single individual’s negligence; rather, they often stem from a chain of contributing factors—team miscommunication, procedural gaps, or institutional constraints. “*In practice, mistakes are rarely individual—they are usually team-based, involving protocols, communication, or system failures. But the public often wants a single person to blame*” (Physician Group 2).

These differing perspectives create a potential trust gap: the public personalizes responsibility, while clinicians operate within a systems-oriented paradigm that diffuses individual blame. For medical students, navigating these expectations involves learning how to communicate candidly about errors while also explaining the collective nature of healthcare delivery. This subtheme illustrates the challenge of reconciling the public’s desire for moral clarity with the realities of shared responsibility in healthcare delivery.

While we positioned Accountability and Responsibility within Theme 2 to reflect participants’ emphasis on individual professional behaviors and moral integrity, we acknowledge its close relationship with the systemic and cultural challenges highlighted in Theme 3. Public participants frequently framed accountability in personal terms—expecting physicians to admit errors openly—whereas physicians often understood responsibility within complex team- or system-based contexts. This tension reflects not only a competency expectation but also a structural reality of Taiwanese healthcare, where professional conduct is continuously negotiated between personal integrity and institutional constraints. For this reason, Subtheme 8 primarily resides under Theme 2 but is also noted as intersecting with the cultural and systemic tensions elaborated in Theme 3.

#### 9. Peer Respect and Teamwork

While largely invisible to the public’s conceptualization of professionalism, physicians and medical students underscored the centrality of respectful collaboration within clinical teams. For many, professionalism extended beyond patient interactions to encompass how colleagues treat one another, particularly in high-pressure environments. As one physician put it, “*Professionalism isn’t just about patients—it’s also about how we treat each other. Mutual respect makes the whole team function better*” (Physician Group 1).

Yet, hierarchical norms in Taiwanese medical culture often constrain open dialogue, especially for those in training. Medical students described the professional risks of challenging a senior’s judgment, even when patient safety might be at stake: “*As a junior, it’s risky to question a senior’s decision, even if you see something wrong. You might be labeled as difficult or disrespectful*” (Medical Students Group 2). These accounts highlight how authority structures can discourage upward communication, potentially undermining the collaborative ethos that underpins safe, high-quality care.

This subtheme reveals teamwork as a “hidden domain” of professionalism—vital for patient safety and clinical efficiency, yet rarely foregrounded in public expectations. It also suggests that cultivating true interprofessional respect requires cultural shifts within institutions, enabling juniors to voice concerns without fear of reprisal. In this way, the professional ideal of teamwork intersects with broader systemic reforms in education and organizational culture, linking individual conduct to the collective functioning of healthcare teams.

**Theme 2 Synthesis**. Across the subthemes in Theme 2, professionalism was constructed as a set of observable skills and behaviors that extend from communication to accountability. Public participants consistently highlighted qualities such as clarity, patience, and honesty as evidence of professionalism, whereas physicians and students emphasized the constraints of clinical workload, systemic complexity, and the realities of error in team-based care. Together, these subthemes reveal that professionalism in Taiwan is conceptualized not only as technical competence and lifelong learning but also as affective and ethical performance in everyday interactions. This theme therefore situates professionalism at the intersection of individual responsibility, relational skill, and systemic accountability.

### Theme 3: Cultural and Systemic Tensions Shaping Professionalism

#### 10. Professionalism as Service Industry vs. Ethical Autonomy

A persistent cultural tension was constructed through analysis around whether physicians should be understood primarily as service providers or as autonomous professionals. Public participants often applied a consumer-service frame, likening medical encounters to retail or hospitality contexts in which courtesy, attentiveness, and deference are paramount. Within this framing, the physician–patient relationship was transactional: patients were “customers,” and their satisfaction was the primary measure of quality. As one participant expressed, “*Physicians should treat patients like customers—they are providing a service. Courtesy and attentiveness come first*” (Public Group 3).

Physicians, however, resisted this analogy, cautioning that it risks subordinating clinical judgment to consumer preference. They emphasized that professionalism is grounded not in customer appeasement but in ethical autonomy—the obligation to act in the patient’s best medical interest, even when doing so requires declining inappropriate or non-beneficial requests. “*We are not waiters. Our duty is to do what’s right, not what’s pleasing. Professionalism means having the courage to say no when it matters*” (Physician Group 5).

This divergence captures a core ethical fault line in contemporary medical culture. It highlights the contrasting ways that professionalism is defined: as service responsiveness by the public and as principled independence by physicians.

#### 11. Family-Centered Decision-Making vs. Individual Autonomy

A central cultural tension in Taiwanese medical professionalism was described in terms of balancing individual consent with family-led decision-making. Public participants overwhelmingly endorsed a family-centered approach, framing it as both morally correct and emotionally protective—especially when serious or life-threatening diagnoses were involved. As one participant explained, “*You must talk to the family first—patients shouldn’t bear this alone. It’s more caring, more responsible*” (Public Group 5).

Physicians acknowledged that their formal training emphasized autonomy and the primacy of patient consent, yet described routinely deferring to family wishes in practice. Such deference was viewed as essential to preserving trust, avoiding confrontation, and aligning with the relational expectations. One physician reflected, “*In theory, autonomy is clear. In practice, family comes first. Ignoring that reality would damage the relationship*” (Physician Group 4).

These accounts underscore that professionalism is not solely the application of abstract, universal principles but the adaptive enactment of ethics within a socio-cultural context. Participants portrayed this as an ongoing negotiation between honoring patient agency and respecting familial authority in clinical practice.

**Theme 3 Synthesis**. The subthemes within Theme 3 underscore that professionalism in Taiwan is continuously negotiated within cultural expectations and systemic pressures. Public participants frequently invoked service-oriented metaphors and family-centered norms, while physicians framed professionalism as the courage to act with ethical autonomy, even when it conflicted with consumerist or familial demands. Together, these accounts illustrate how professionalism is shaped by the interplay of societal expectations, healthcare system structures, and professional judgment.

### The Divergent Emphases

A thematic matrix (Table 2) illustrates the interpretive divergences across stakeholder groups. The findings suggest that medical professionalism in Taiwan is not a singular or static construct, but rather a fluid, culturally negotiated set of values and behaviors shaped by social roles, institutional norms, and everyday clinical realities.

**Table 2:** Divergent Constructions of Medical Professionalism Across Public and Clinical Stakeholder Perspectives in Taiwan.


THEME	SUBTHEME	PUBLIC PERSPECTIVE	PHYSICIAN/INTERN PERSPECTIVE

Core Values Underpinning Professionalism	1. Humanism-Based Care	Warmth and moral virtue define a good doctor.	Compassion is essential, but emotional boundaries are necessary to prevent burnout.

	2. Family-Centered Empathy	Family inclusion in decision-making is a sign of respect and professionalism.	Ethical tension between cultural norms and patient autonomy as taught in training.

	3. Moral Character and Role Modeling	Physicians should serve as ethical exemplars in society.	Role modeling by seniors is more influential than formal professionalism instruction.

Expected Competencies and Behaviors	4. Communication Skills	Friendly tone, patience, and clarity are central to professionalism.	Communication must balance empathy with time constraints and clinical efficiency.

	5. Emotional Labor	Professionals should always show emotional availability, regardless of fatigue.	Empathy fatigue is a major challenge; emotional labor is often invisible and draining.

	6. Honesty and Transparency	Full disclosure is a moral duty, even for bad news.	Selective truth-telling is sometimes necessary to protect patients or avoid conflict.

	7. Technical Competence and Lifelong Learning	Competence is assumed; humanistic care distinguishes professionals.	Continuous learning is a core professional obligation in an evolving medical field.

	8. Accountability and Responsibility*	Physicians should personally own up to errors.	Responsibility is often shared across teams; systemic issues contribute to mistakes.

	9. Peer Respect and Teamwork	Rarely discussed; professionalism seen as primarily patient-facing.	Respect across hierarchies is vital, but juniors often hesitate to challenge seniors.

Cultural and Systemic Tensions Shaping Professionalism	10. Service Industry vs. Ethical Autonomy	Physicians should act like service providers and accommodate patient demands.	Physicians prioritize ethical autonomy over customer-service expectations.

	11. Family vs. Individual Autonomy	Family-first decision-making protects patients and reflects cultural norms.	Family involvement is culturally expected but can conflict with legal and ethical norms.


*Note: Although positioned under Theme 2, this subtheme also intersects with Theme 3, as accountability is shaped not only by individual professional behaviors but also by broader cultural expectations and systemic realities.

As summarized in Table 2, the findings reveal that professionalism in Taiwan is not a fixed or universally agreed-upon construct but a socially negotiated ideal shaped by cultural ethics, institutional contexts, and the relational positioning of different stakeholder groups. Public participants consistently foregrounded humanism-based care and family-centered empathy (Theme 1, Subtheme1–2) as moral imperatives, often framed through service-oriented metaphors. Physicians and medical students, while valuing these same principles, reinterpreted them through the lens of emotional sustainability (Theme 2, Subtheme 5), ethical autonomy (Theme 3, Subtheme 10), and the realities of technical competence and lifelong learning (Theme 2, Subtheme 7). These divergent emphases demonstrate that shared values—such as compassion, accountability, and integrity—are operationalized differently depending on whether one occupies the role of care recipient, trainee, or experienced clinician.

Across themes, professionalism in Taiwan was constructed as a socially constructed, culturally embedded concept shaped by the interplay of public expectations, professional self-concept, and institutional realities. Public participants articulated clear moral and behavioral expectations—emphasizing courtesy, emotional availability, family-centered care, and visible role modeling—which often served as the reference point for judging physicians’ conduct. Physicians and medical students, while sharing many of these values, framed professionalism through the lens of ethical autonomy, technical competence, and navigating systemic constraints. Points of convergence, such as valuing honesty and respect, coexisted with tensions over the limits of patient sovereignty, the role of family in decision-making, and the balance between service orientation and professional independence. These findings suggest that professionalism is not a static list of traits, but a negotiated social practice in which the public is an active participant. Recognizing this reciprocal dynamic highlights the importance of engaging both healthcare professionals and the public in cultivating a shared, contextually relevant understanding of what it means to be “professional” in Taiwanese medicine. This reciprocal dynamic highlights the importance of engaging both healthcare professionals and the public in cultivating a shared understanding of professionalism that is responsive to Taiwan’s cultural and institutional context.

## Discussion

This study explored how medical professionalism is defined, enacted, and expected across three key stakeholder groups in Taiwan — practicing physicians, final-year medical students, and members of the public — as part of a purposeful triangulated design. This multi-stakeholder approach was theoretically informed by social constructionism [27,28], which conceptualizes professionalism as a negotiated moral and social contract between the profession and society. By comparing the perspectives of physicians, medical students, and the public, we were able to illuminate not only points of consensus but also culturally specific tensions and interpretive divergences that would remain hidden in single-group studies. Thematic analysis revealed eleven culturally embedded themes organized into three themes: core values, behavioral expectations, and cultural-systemic tensions. Together, these findings illustrate that professionalism in Taiwan is deeply relational, shaped by Confucian ethics, evolving social expectations, and the institutional dynamics of the National Health Insurance (NHI) system [10,11]. Professional identity formation in this context involves continuous negotiation across clinical competence, moral character, emotional availability, and family-based social roles. Crucially, our findings reaffirm a disjunction between Western professionalism frameworks—centered on individual autonomy, accountability, and regulatory norms—and Taiwanese societal expectations that prioritize collective harmony, relational obligation, and family-centered decision-making. These tensions, also documented in Confucian-influenced societies such as China and Korea [8,13,15], underscore the limitations of assuming professionalism as a culturally neutral construct. In relation to existing literature, our findings both corroborate and extend previous scholarship on professionalism in East Asian and global contexts. Consistent with Fan [10] and Ho [17], we confirm the enduring influence of relational ethics, family-centered care, and hierarchical harmony on professional norms in Taiwan. Our results also align with Zhu et al. (2022) in highlighting the hidden curriculum’s role in transmitting these values. At the same time, our analysis challenges the presumed universality of Western-centric professionalism frameworks by showing how principles such as patient autonomy and full disclosure are negotiated in culturally specific ways that privilege familial authority and emotional protection [3,19]. Newly illuminated in this study is the framing of emotional labor as a central, culturally embedded dimension of professionalism; the active role of public expectations in shaping moral norms; and the transitional function of medical students as interpreters between formal curricular ideals and societal expectations. These insights contribute a nuanced, multi-stakeholder perspective to the international discourse on culturally responsive professionalism.

Our study advances the field in several ways. First, it affirms a growing body of critical scholarship that challenges the universality of Western professionalism models [7,9,17]. While earlier studies have examined student or educator perspectives, ours triangulates insights from physicians, medical students, and members of the public, revealing a dynamic, co-constructed understanding of professionalism as a negotiated moral and social relationship [27,28]. Second, we underscore the hidden curriculum’s pivotal role in socializing students into local norms that diverge from formal curricula. Though Taiwan’s medical education system nominally endorses bioethical standards such as informed consent and impartial truth-telling [4,6], students internalize professionalism primarily through observation and role modeling [19,21]. This informal learning emphasizes hierarchical respect, strategic communication, and family-first decision-making, patterns also seen across Asia [18,22]. Third, our findings draw attention to emotional labor as a core but underrecognized dimension of professionalism. Participants described the psychological demands of maintaining empathy, patience, and emotional restraint under time pressure and high caseloads. Such expectations, amplified by a service-oriented healthcare ethos, contribute to identity strain and burnout, echoing broader concerns in the literature on moral distress in Confucian cultural contexts [12,14].

A key insight is that professionalism dilemmas are not confined to the early stages of training. Even experienced physicians reported adapting their moral reasoning and communicative practices to balance institutional mandates with culturally grounded expectations. These adjustments suggest that professionalism should be understood as a lifelong adaptive process embedded in shifting ethical, relational, and organizational terrains [25,28]. Equally important is the public’s role in shaping the moral contours of professionalism. Under Taiwan’s NHI, patients and families expect high-quality care delivered with humility, respect, and emotional attentiveness. These expectations often position the physician as a service provider, which clashes with professional self-conceptions centered on ethical judgment and clinical autonomy [16,31]. As such, professionalism is judged not only through behavior but also through affective presentation, further complicating the terrain of physician identity [18].

We propose a relational professionalism framework grounded in constructivist and sociocultural theory. This model views professionalism as a context-sensitive, interactional process shaped by interpersonal roles, institutional constraints, and culturally specific moral logics [27,32]. Rather than framing professionalism as a universal internal trait, this approach situates professional identity in dynamic, ethically charged relationships among stakeholders. This perspective aligns with theories of relational autonomy, which reject methodological individualism in favor of embedded, interdependent moral agency [7,12]. In East Asian societies shaped by Confucianism, values such as filial piety, ritual propriety, and moral character are not peripheral to medical professionalism—they are central [8,13].

### Practical Implications for Medical Educators

Medical educators should move beyond abstract, universalistic models of professionalism and engage with the lived moral worlds of learners, patients, and the public. Because this study included the public as a stakeholder, professionalism education should also address the reciprocal role of public expectations in shaping doctor–patient relationships. Professionalism is co-constructed, and part of the challenge lies in societal expectations of “what a good doctor should be.” Educators should therefore consider not only how to train doctors to meet patient needs, but also how to inform and engage the public in understanding professional boundaries, ethical constraints, and clinical realities. Curriculum design must integrate culturally salient cases—such as navigating family dynamics, deferring to hierarchical authority, and managing emotional labor—while also addressing the reciprocal role of public expectations. This could include community-facing components, such as patient education workshops, public dialogues, and media campaigns to align expectations and build mutual understanding. Reflective practices such as narrative writing, facilitated discussions, and arts-based pedagogy offer promising tools for fostering culturally responsive professionalism [18,33]. Assessment must also evolve [3,19]. Conventional tools prioritize punctuality, etiquette, and communication clarity but fail to capture students’ ability to ethically navigate competing cultural and institutional demands [19,21]. Multi-source feedback and culturally validated empathy scales (e.g., those adapted in Malaysia and Taiwan) offer more context-sensitive alternatives [9,18]. Equally, faculty development is vital [3,18,21]. Medical educators need support in modeling professionalism as situated ethical practice rather than prescriptive rule-following. Programs that encourage reflection on personal values, moral dilemmas, and the hidden curriculum can help cultivate ethically attuned teaching [22,34]. With the focus on the Taiwanese context and the inclusion of multiple stakeholders, these findings offer lessons on how (Western) professionalism frameworks can be adapted and translated across cultural settings, providing insights into the cross-context transferability and limits of dominant professionalism models. Framing professionalism as a mutual, culturally responsive commitment—encompassing both professional conduct and public understanding—may help reduce tensions and enhance trust across stakeholder groups.

### Strengths and Limitations

While our study was conducted in a predominantly Hoklo cultural context, Taiwan’s broader sociocultural landscape also includes Hakka, Indigenous, and immigrant communities, each of which may hold distinct perspectives on professionalism. This diversity suggests that culturally grounded professionalism frameworks should be interpreted with sensitivity to intergroup differences. At the same time, our findings support the view that individual differences in values, professional experiences, and personal identity can transcend cultural boundaries, underscoring the need for professionalism training and policy to remain adaptable to both cultural and individual variation. This study’s strengths include its triangulated design involving three stakeholder groups, which provided a nuanced, multi-perspective account of professionalism in Taiwan, and the use of an interpretivist, constructivist approach that illuminated the cultural and relational dimensions of professional identity. However, several limitations should be noted. First, recruitment was limited to a single tertiary teaching hospital in northern Taiwan and its surrounding community. While this site was chosen for its diverse clinical services and large catchment population, the organizational culture and regional context may differ from other settings, potentially limiting transferability. Second, although purposive sampling achieved diversity in age, gender, and specialty representation within each stakeholder group, the findings may not capture the full range of perspectives present in Taiwan’s varied healthcare environments. Third, focus group dynamics may have influenced how participants expressed their views, particularly on sensitive topics such as moral conduct and hierarchical relationships. Fourth, while Confucian ethics provide an important cultural frame for interpreting our findings, they do not represent all perspectives in Taiwan. Other sociocultural groups may draw on different traditions, which could shape distinct understandings of professionalism. Fifth, ethnic background data were not collected, in accordance with IRB confidentiality guidelines for small focus group research, which precluded analysis of potential differences by ethnicity. Future research should purposively include participants from these diverse ethnic backgrounds to examine how ethnicity shapes professionalism norms and expectations. These limitations should be considered when interpreting our results and applying them to other contexts.

### Unanswered Questions and Future Research

This study opens multiple avenues for future inquiry. First, how do learners’ understandings of professionalism evolve longitudinally across training stages? Second, are there intergenerational shifts in cultural expectations, particularly as younger patients are socialized into more globalized norms? Third, comparative research across Confucian societies—such as Korea, Japan, and China—could clarify regional similarities and distinctions in professionalism. Finally, intervention studies evaluating culturally responsive curricula and reflective pedagogy would help translate theoretical insights into practical change.

## Conclusion

Our study shows that professionalism in Taiwan is not a static or transferable skillset, but a relational and culturally co-constructed process. It is enacted through a complex balancing of technical competence, moral character, emotional attunement, and family-centered ethics. These findings highlight the importance of recognizing both shared values and divergent emphases across stakeholder groups. In conclusion, professionalism should be understood as a dynamic, contextually embedded construct. By foregrounding cultural negotiation, our study underscores the need for professionalism education that is responsive to local moral expectations while attentive to global standards. Such an approach prepares future physicians to navigate cultural complexity with humility, flexibility, and moral integrity.

## Data Accessibility Statement

The dataset supporting the conclusions of this article is included within the article.
